# The Acute Effect of Foam Rolling and Vibration Foam Rolling on Drop Jump Performance

**DOI:** 10.3390/ijerph18073489

**Published:** 2021-03-27

**Authors:** Wei-Chi Tsai, Zong-Rong Chen

**Affiliations:** 1 Division of Physical Medicine and Rehabilitation, Zuoying Branch of Kaohsiung Armed Forces General Hospital, Kaohsiung 813, Taiwan; william-tsai@yahoo.com.tw; 2 Department of Physical Education, National Taiwan Normal University, Taipei 106, Taiwan; 3 Department of Athletic Performance, National University of Kaohsiung, Kaohsiung 811, Taiwan

**Keywords:** warm-up, self-massage, volleyball athletes, foam-roller

## Abstract

The purpose of this study was to examine the acute effect of foam rolling and vibration foam rolling on drop jump performance. The optimal time interval between warm-up using foam rolling or vibration foam rolling and drop jump performance was identified. This study included 16 male NCAA Division I college volleyball athletes. Three interventions were performed in a randomized order: the foam rolling exercise (FRE), vibration foam rolling exercise (VFRE), and static rest (control). The drop jump was performed before interventions, as well as 2 and 5 min after interventions. The FRE exhibited higher values for drop jump height (DJH) (*p* = 0.001; η^2^ = 0.382; statistical power = 0.964) and mean power generation at the hip joint (*p* = 0.006; η^2^ = 0.277; statistical power = 0.857) at 2 min compared with before intervention but not at 5 min (*p* > 0.05). However, the VFRE showed no significant changes in DJH (*p* > 0.05), and found that hip_power_ was decreased at 5 min (*p* = 0.027; η^2^ = 0.214; statistical power = 0.680). The FRE completed in 2 min before rapid single action competition (sprint, long jump, triple jump, etc.) could increase sports performance.

## 1. Introduction

The fast stretch-shortening cycle (FSSC) performance has important practical implications because many competitive sports require rapid movement performance, such as agility, sprinting, and approach jumping [1]. The drop jump (DJ) is often used to examine FSSC performance, which involves dropping down from a box and performing a vertical jump as quickly as possible after landing [1,2]. Recently, it has been suggested that high-intensity muscle contraction conditions, such as resistance exercise [3,4], plyometric exercise [1], and whole-body vibration [5] as a warm-up protocol produce a subsequent increase in FSSC performance. This phenomenon has been called post-activation performance enhancement (PAPE) [6,7]. However, reviews of studies have also indicated that the practical applicability problem of PAPE was limited due to required equipment in a practical setting [7,8]. Based on the above, PAPE is inapplicable at the site of competition or training.

In recent years, foam rolling exercise (FRE) and vibration foam rolling exercise (VFRE) have been used as a new warm-up option that is highly regarded by coaches, conditioning professionals, and athletes [9] because of their convenience and ease of practice in the various sites of competition or training [10,11]. FRE and VFRE are self-massage techniques performed using foam and vibrating foam rollers, respectively, rather than a massage by a physical therapist or trainer [9,12]. Most studies have found no effect on countermovement jump (CMJ) and squat jump performance after FRE [10,13,14,15], but other studies found an increased CMJ performance after FRE [14,16]. The FSSC requires more elastic energy contribution than a slow stretch-shorting cycle (SSSC) [2]. Hence, these results suggested that the FRE will likely produce greater positive effects in the FSSC than in the SSSC, based on the assumption that the FRE can induce moderate muscle compliance due to increased muscle temperature induced by prolonged rolling, which increases musculotendinous unit storage and/or generates elastic energy [14,17]. Furthermore, if the VFRE induces vibration stimulation and enhances neural potentiation by tonic vibration reflex as described by Lamont, et al. [18], then the VFRE could be expected to show greater improvements on FSSC than the FRE [19]. Previous studies have found that neither FRE nor VFRE affects CMJ performance [20], but another study found that both the FRE and VFRE had similar benefits on CMJ performance [21]. These studies have only focused on the effects of the FRE and VFRE on SSSC, such as CMJ performance; no known published studies have examined the acute effects of the FRE and VFRE on FSSC (i.e., DJ).

It is crucial to determine the optimal time interval between completing the warm-up protocol and competition initiation. It would allow coaches and conditioning professionals a time reference for improving sports performance. Previous studies have indicated the time after an acute bout of FRE and VFRE and collected multiple testing measures at individual time points [11,14,22], including flexibility [11,20,22], vertical jump [11,14,22], muscle strength [11,22], agility tests [10] joint proprioception [9], 10 min running test [14], and balance [9]. However, these studies lack the optimal time interval between post-FRE or post-VFRE and competition.

Therefore, this study aimed to investigate the acute effect of FRE and VFRE on DJ performance and to determine the optimal time interval between post-FRE or post-VFRE and competition. Based on the previous literature, we hypothesized that FRE would subsequently increase DJ performance, and VFRE would improve DJ performance more than FRE.

## 2. Materials and Methods

### 2.1. Subjects

The study included 16 male NCAA Division I college volleyball players (177.56 ± 5.05 cm, 69.37 ± 6.66 kg, 21.5 ± 1.15 years). Inclusion criteria included having more than 5 years of experience in plyometric and resistance training. In contrast, exclusion criteria included lower extremity surgery, respiratory disease, catastrophic illness, or lower extremity injury within the past 6 months, as these conditions could affect normal DJ motion. The subject was instructed to maintain a normal diet and to abstain from medications, alcohol, and additional supplementation throughout the study. Before completing the testing protocol, all the subjects read and signed informed consent document. The Antai Medical Care Corporation Institutional Review Board approved this study (File number 19-011-B).

### 2.2. Design and Procedures

**Figure 1:**
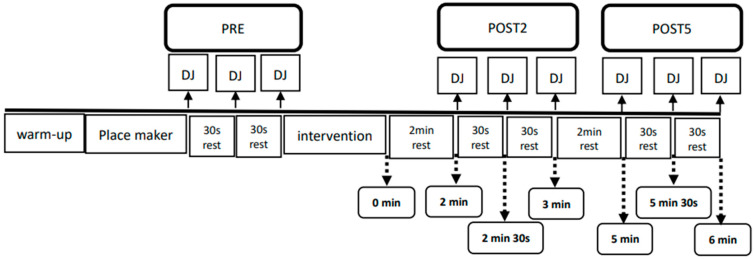
Schematic diagram of the experimental procedures.

In this randomized within-subject study design, subjects reported to the laboratory for 4 separate sessions, separated by 24 h between them. During the first session, subjects were informed of the benefits and risks of the investigation, informed consent documents were collected, and body mass and height data were obtained. Moreover, the subjects were taught how to perform DJ and FRE and were familiarized with all the testing procedures. During the second, third, and fourth sessions, all subjects wore tight shorts and identical shoes of the same brand with their appropriate size (Maximizer16; Mizuno Taiwan Corporation, Taipei, Taiwan) and then performed 5 min of light aerobic jogging as a warm-up. The 21 reflective markers were placed bilaterally over the anterior-superior iliac spines, posterior–superior iliac spine, anterior thighs and shank, lateral and medial knee, lateral and medial ankle, heel, and the fourth metatarsal heads; the sacrum received only one marker. All reflective markers were measured using a three-dimensional motion analysis system (Motion Analysis Corporation, Santa Rosa, CA, USA). Immediately after the reflective markers were placed, the subjects performed 3 DJs from 40 cm as a pre-test (PRE). The rest period between each jump was 30 s. The subjects were instructed to keep their hands on their hip and to drop from a 40-cm-high box onto two force plates. They were instructed to perform a maximal vertical jump as quickly as possible after ground contact, maintaining the hips, knees, and ankles in a fully extended position throughout the entire jump [23]. The subjects performed control (Con), VFRE, and FRE randomized for the second, third, and fourth sessions. For the Con, the subjects were instructed to sit on a chair for 15 min of passive rest. However, the subjects performed the VFRE using a vibrating foam roller constructed with a vibrating generating motor center surrounded by polypropylene (Vyper By Hyperice 2.0, Irvine, CA, USA). The VFRE was applied to eight muscle regions in the bilateral lower limb in the following order: quadriceps with flexed knee, quadriceps with extended knee, gluteus, biceps femoris, tibialis anterior muscle, gastrocnemius, fasciae latae, and plantar fascia. First, the subject rolled a muscle region in the right lower limb; subsequently, the same muscle region in the left lower limb was rolled. Each unilateral muscle region was rolled for 1 min. Bilateral plantar fasciae were rolled simultaneously for 1 min. The total VFRE time was 15 min. The subjects were instructed to put their body weight on the vibrating foam roller (of 45 Hz) as much as possible. The rolling frequency was set at 40 beats per minute (bpm) using an electronic metronome. Each rolling cycle (distal to proximal and return) was about 3 s. The entire process was supervised by a strength and conditioning coach. This protocol was based on previous studies’ reports [9,22]. The high frequency (45 Hz) of VFRE was used because the participants were elite volleyball players. The FRE employed the same method for the VFRE, except that the vibrating motor was turned off. This study used the same roller in both protocols to control varying surface levels in different foam rollers. Post-test measures were performed in the same manner as the PRE measure at 2 (POST2) and 5 (POST5) minutes after the intervention. The 3 DJs (POST2) were performed between 2 and 3 min after intervention. The 3 DJs (POST5) were performed between 5 and 6 min after intervention. A schematic diagram of the experimental procedure is shown in Figure 1.

### 2.3. Instrumentation

Kinetic and kinematic data were simultaneously captured by two force platforms (AMTI Inc., Watertown, MA, USA) (2000 Hz) and a 10-camera three-dimensional (3D) motion analysis system (Motion Analysis Corporation, Santa Rosa, CA, USA) (200 Hz). The right leg data was collected by one force platform, while the left leg data was collected by another force platform. The EVaRT (Version 4.6, Motion Analysis Corporation, Santa Rosa, CA, USA) software was used to record the kinetic and kinematic data.

### 2.4. Data Analyses

**Figure 2:**
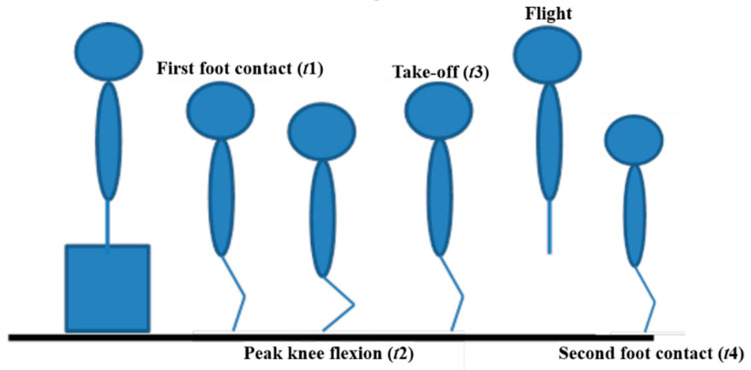
Illustrates the phases of DJ.

All raw kinetic and kinematic data from the dominant leg were analyzed using custom MATLAB software (The MathWorks, Inc., Natick, MA, USA). The dominant leg was defined as the leg regularly used to kick a ball [24]. The first ground contact phase was defined as the instance between the first foot contact after drop-off from a 40 cm box and take-off of the foot from the force platforms. The first ground contact phase was divided into the landing and take-off subphases. The landing subphase was described as the period from the instant of first foot contact (*t*_1_) to peak knee flexion (*t*_2_) and the take-off subphase as the period from peak knee flexion (*t*_2_) to the take-off of the foot from the force platforms (*t*_3_). Additionally, the flight subphase was defined as the period from the take-off of the foot from the force platforms (*t*_3_) to the instant of second foot contact (*t*_4_). Figure 2 illustrates the phases of the DJ. The instant of the first and second foot contact (*t*_1_ and *t*_4_) and take-off of the foot from the force platforms (*t*_3_) was determined by assessing the 30 Newton vertical ground reaction force threshold. 

The formula: $\frac{1}{8}$*g*T^2^ (g = 9.81 m/s^−2^; T = *t*_4_ − *t*_3_) was used to calculate drop jump height (DJH) [25,26]. The mechanical power of the hip, knee, and ankle joints was calculated by inverse dynamics and normalized by body weight. The joint power generated by the joint muscle and tendon was defined as positive mechanical power [26]. The joint power was averaged in the take-off subphase. The following formula was used to calculate reactive strength index (RSI) of DJ: $\frac{DJH}{t3\;-\;t1}$. The ground contact time (GCT) was calculated by the time between *t*_1_ and *t*_3_. The representative value of each dependent variable was calculated by averaging the data obtained from the three trials.

### 2.5. Statistical Analyses

Data were analyzed using SPSS software (Version 12.0, SPSS Inc., Chicago, IL, USA). All results are reported as mean ± standard deviation (SD). All dependent variables were analyzed using a two-way repeated-measures analysis of variance (3 time points [PRE vs. POST2 vs. POST5] × 3 protocols [CON vs. VFRE vs. FRE]) followed by Bonferroni post hoc analysis. The significance level was set at *p* ≤ 0.05. The partial eta squared was classified using the following scale: small = 0.01, medium = 0.06, large = 0.15 [27]. The intraclass correlation coefficient (ICC) was computed for PRE, POST2, and POST5 (each protocol) for all variables. The ICC was classified using the following scale: values less than 0.4 (poor), between 0.40 and 0.59 (fair), between 0.60 and 0.74 (good), and between 0.75 and 1.00 (excellent) [28]. The coefficient of variation (CV) was calculated across three PRE measurements [29,30]. The acceptable CV was below 10.8% [29].

## 3. Results

The DJH was found to have excellent reliability at PRE, POST2, and POST5 in all the three protocols (ICC = 0.791–0.929). The CV was 10% for DJH. The DJH time × protocol interaction was significant (*p* = 0.022; η^2^ = 0.171; statistical power = 0.781). Simple main effects showed significant improvements in DJH after FRE at POST2 when compared with PRE (*p* = 0.001; η^2^ = 0.382; statistical power = 0.964), but the Con and VFRE were not significant (*p* > 0.05). In addition, simple main effects showed no significant difference between Con, VFRE, and FRE at PRE (*p* > 0.05) (Table 1).

The RSI was found to have good reliability at PRE, POST2, and POST5 in all the three protocols (ICC = 0.642–0.780). The CV was 9.3% for RSI. The time × protocol interaction for RSI was significant (*p* = 0.029; η^2^ = 0.163; statistical power = 0.753) (Table 2). However, both FRE (*p* = 0.000; η^2^ = 0.539; statistical power = 0.995) and Con (*p* = 0.005; η^2^ = 0.298; statistical power = 0.868) showed that POST2 and POST5 were higher than PRE, and the FRE showed that POST2 was higher than POST5. The VFRE was found higher than FRE and Con at PRE (*p* = 0.003; η^2^ = 0.358; statistical power = 0.945), and higher than FRE at POST5 (*p* = 0.026; η^2^ = 0.215; statistical power = 0.683) (Table 2).

The GCT was found to have good reliability at PRE, POST2, and POST5 in the three protocols (ICC = 0.877–0.917). The CV was 7.2% for GCT. For ground contact time, the time × protocol interaction was not significant (*p* > 0.05). A significant main effect was seen for time (*p* = 0.000; η^2^ = 0.452; statistical power = 0.992). The Con, VFRE, and FRE showed that POST2 and POST5 were lower than PRE, and POST2 was lower than POST5. A significant main effect was seen in the protocol (*p* = 0.003; η^2^ = 0.328; statistical power = 0.912). The VFRE was lower than Con (Table 3).

The hip_power_ was between fair to good reliability at PRE, POST2, and POST5 in the three protocols (ICC = 0.542–0.973). The CV was more than 10.8% for hip_power_. The time × protocol interaction was significant for the hip_power_, (*p* = 0.000; η^2^ = 0.317 statistical power = 0.991). Simple main effects showed significant improvements in the hip_power_ after FRE at POST2 when compared with PRE (*p* = 0.006; η^2^ = 0.277 statistical power = 0.857), but the VFRE showed that POST5 was lower than PRE (*p* = 0.027; η^2^ = 0.214; statistical power = 0.680). In addition, simple main effects showed no significant difference between Con, VFRE, and FRE at PRE (*p* > 0.05) (Table 4).

The knee_power_ was between fair to excellent reliability at PRE, POST2, and POST5 between the three protocols (ICC = 0.558–0.928). The CV was more than 10.8% for knee_power_, and the time × protocol interaction was significant (*p* = 0.047; η^2^ = 0.147 statistical power = 0.692). However, simple main-effects testing failed to identify significant pairwise differences among time and protocols (*p* > 0.05) (Table 5).

The reliability of ankle_power_ at PRE, POST2, and POST5 was between fair to excellent in the three protocols (ICC = 0.593–0.826). The CV was more than 10.8% for ankle_power_, and the time × protocol interaction was not significant (*p* > 0.05). (Table 5). A significant main effect was seen for protocol (*p* = 0.032; η^2^ = 0.205; statistical power = 0.655). The VFRE was higher than Con (Table 6).

## 4. Discussion

This study aimed to compare the acute effect of FRE versus VFRE on DJ performance and to determine the optimal time interval between post-FRE or post-VFRE and competition. The present results show that the FRE significantly increased DJH and hip_power_ at POST2; however, there was no significant change at POST5. Furthermore, no significant changes in DJH, and hip_power_ were decreased at 5 min after VFRE. In terms of RSI and GCT, no significant improvements were observed for FRE and VFRE at POST2 and POST5. We hypothesized that both FRE and VFRE would subsequently increase DJ performance, and VFRE would produce more increase in DJ performance than that of FRE. This initial hypothesis was partly supported by the present result.

This study found that the DJH was increased after FRE. This study confirmed that when 60 s FREs were performed for each unilateral muscle region, this protocol can increase DJH. This result might be explained by the fact that the FRE can induce moderate muscle compliance to increase the ability of the musculotendinous unit to store and/or generate elastic energy [14,17]. In contrast, most studies found that the FRE had no effect on SSSC muscle performance, such as CMJ and SJ [10,13,14,15]; only a few studies found a positive effect on SSSC [14,16], and this may be attributed to the requirement of more storage and generation of elastic energy in DJ than CMJ and SJ; thus, the FRE was limited in increasing SSSC muscle performance [2].

The RSI was influenced by DJH and GCT and was often used to examine jump performance during a rapid jump movement [26]. In the current study, even though the RSI was not improved after FRE, the GCT was unchanged, and DJH was increased when examining the two components of RSI separately. This means that the FRE-increased-DJ is a quick movement with very short (0.297–0.304 s) GCT. Besides, the GCT may be mainly influenced by the jumping strategies adopted [31]. Specifically, all subjects were instructed to perform a maximal vertical jump as quickly as possible after ground contact during DJ.

The present investigation confirms that FRE simultaneously performed with local vibration (VFRE) could not influence DJH. While the authors of this study are not aware of another VFRE study that examines DJ performance, this study’s result was consistent with results reported in previous investigations which reported that 5 min VFRE did not affect CMJ performance [20]. However, compared to previous studies, it was found that 30 s VFRE can increase CMJ performance [21]. It seems reasonable to speculate that 60 s VFRE is an excessive duration to induce fatigue. Specifically, the vibration stimulation not only induces PAPE by neural potentiation but also induces fatigue, which was dominant after vibration stimulation, negatively affecting subsequent performance [18]. Besides, the hip_power_ was decreased at 5 min after VFRE. This implied that the fatigue wound influences the hip mechanism adaptation.

Previous studies have indicated that moderate muscle compliance was probably enhanced by the FRE [14,32]. The underlying physiological mechanism of this adaptation may be due to the acutely altered balance between the viscous and elastic property of the fascia; the fascia becomes more elastic through heat from rolling friction in FRE [14,32]. This physiological mechanism has contributed to the change in biomechanical properties that resulted in better power generation of the hip [14,32]. This implies that increasing the hip_power_ is one of the main factors associated with increased DJH after FRE [33]. However, this study found that knee_power_ and ankle_power_ were not influenced by the FRE. This result may be due to the gluteus being rolled while in a sitting position which resulted in more pressure than other muscle groups and induced more moderate muscle compliance than in other muscle groups.

This study found that FRE significantly increased DJH at POST2 when compared with PRE. However, this improvement was not maintained at POST5. Although other investigators have evaluated different times and found positive effects on CMJ performance [14,16], those studies did not provide an accurate time interval between post-FRE and competition. Our results are important because this study established a 2-min interval time between post-FRE and competition. Furthermore, this study also found that the hip_power_ increased at 2 min after FRE, but not at 5 min. This would imply that the elasticity properties of the muscle and connective tissue were increased at 2 min after FRE; however, this benefit dissipated at 5 min after FRE. This may help explain why DJH was increased at POST2 but not at POST5.

This study had four limitations. First, the subjects were male college volleyball players and elite athletes, which inhibits the generalizability of the results to the population; future studies should examine other populations, such as female and teen-aged elite athletes. Second, the testing measurement is the DJ; future studies should examine other FSSC testing measurements, such as the sprint, approach jump, and agility. Third, the lack of ultrasound did not allow us to examine the acute effect of FRE on the connective tissue characteristics. Fourth, the lack of electromyography did not allow us to examine the different mechanisms behind the FRE and VFRE.

## 5. Conclusions

The findings of the present study demonstrate that DJH and hip_power_ were increased at 2 min after FRE in elite athletes, although this improvement cannot be extended to 5 min. However, there was no significant difference in DJH, and hip_power_ was decreased at 5 min after VFRE. Based on these findings, the FRE can improve subsequent competitive sports performance that requires rapid movement. This study suggests the athletes of rapid movement single action, such as in the 100 m race, long jump, triple jump etc., who complete the protocol 2 min before the competition might benefit from the FRE.

## Figures and Tables

**Table 1:** The DJH of DJ (cm); mean ± SD †.

	PRE	POST2	POST5
Con	28.29 ± 5.86	28.38 ± 6.05	28.67 ± 5.73
VFRE	28.26 ± 5.10	27.96 ± 5.58	28.14 ± 5.30
FRE	26.68 ± 5.66	27.82 ± 5.29 *	27.16 ± 5.45

**†** = interaction (time × protocol); * = compare to PRE (*p* < 0.05).

**Table 2:** The RSI of DJ (cm·s^−1^); mean ± SD **†**.

	PRE	POST2	POST5
Con	90.46 ± 15.33	95.52 ± 18.40 *	93.82 ± 17.39 *
VFRE	100.16 ± 13.96 ^a^	100.34 ± 16.94	101.16 ± 16.38 ^b^
FRE	89.53 ± 16.88	95.90 ± 16.55 *^,#^	92.88 ± 16.44 *

**†** = interaction (time × protocol) (*p* < 0.05); **^a^** = compare to Con and FRE at PRE (*p* < 0.05); **^b^** = compare to FRE at POST5 (*p* < 0.05); * = compare to PRE (*p* < 0.05); ^#^ = compare to POST5 (*p* < 0.05).

**Table 3:** The GCT of DJ (s); mean ± SD **+**.

	PRE	POST2	POST5
Con	0.321 ± 0.081	0.306 ± 0.078 *^,#^	0.316 ± 0.083 *
VFRE ^‡^	0.287 ± 0.066	0.283 ± 0.068 *^,#^	0.284 ± 0.068 *
FRE	0.304 ± 0.066	0.297 ± 0.064 *^,#^	0.299 ± 0.063 *

**+** = significant main effect for time and protocol (*p* < 0.05); **^‡^** = difference was found between VFRE and Con (*p* < 0.05); * = compare to PRE (*p* < 0.05); ^#^ = compare to POST5 (*p* < 0.05).

**Table 4:** The hip_powe_ of DJ (W/BW); mean ± SD **†**.

	PRE	POST2	POST5
Con	0.059 ± 0.065	0.066 ± 0.071	0.063 ± 0.075
VFRE	0.058 ± 0.047	0.050 ± 0.037	0.050 ± 0.042 *
FRE	0.062 ± 0.052	0.078 ± 0.064 *	0.068 ± 0.056

**†** = interaction (time × protocol) (*p* < 0.05); * = compare to PRE (*p* < 0.05).

**Table 5:** The knee_power_ of DJ (W/BW); mean ± SD **†**.

	PRE	POST2	POST5
Con	0.01 ± 0.06	0.004 ± 0.06	0.008 ± 0.06
VFRE	0.02 ± 0.04	0.02 ± 0.04	0.02 ± 0.04
FRE	0.03 ± 0.05	0.02 ± 0.06	0.03 ± 0.06

**†** = interaction (time × protocol) (*p* < 0.05).

**Table 6:** The ankle_power_ of DJ (W/BW); mean ± SD **+**.

	PRE	POST2	POST5
Con	0.58 ± 0.21	0.64 ± 0.23	0.62 ± 0.23
VFRE ^‡^	0.69 ± 0.28	0.69 ± 0.28	0.70 ± 0.28
FRE	0.61 ± 0.25	0.62 ± 0.27	0.61 ± 0.25

**+** = significant main effect for protocol (*p* < 0.05); **^‡^** = difference was found between VFRE and Con (*p* < 0.05).

## Data Availability

The data presented in this study are available on request from the corresponding author. The data are not publicly available due to privacy and ethical restrictions.
